# Fused Filament Fabrication-4D-Printed Shape Memory Polymers: A Review

**DOI:** 10.3390/polym13050701

**Published:** 2021-02-26

**Authors:** Sara Valvez, Paulo N. B. Reis, Luca Susmel, Filippo Berto

**Affiliations:** 1 C-MAST, Department of Electromechanical Engineering, University of Beira Interior, Calçada Fonte do Lameiro, 6201-100 Covilhã, Portugal; sara.valvez@ubi.pt; 2 Department of Civil and Structural Engineering, The University of Sheffield, Mappin Street, Sheffield S1 3JD, UK; l.susmel@sheffield.ac.uk; 3 Department of Industrial and Mechanical Engineering, Norwegian University of Science and Technology, Richard Birkelands vei 2b, 7491 Trondheim, Norway; filippo.berto@ntnu.no

**Keywords:** additive manufacturing (AM), fused filament fabrication (FFF), 4D printing, shape memory effect (SME), mechanical performance, structural integrity

## Abstract

Additive manufacturing (AM) is the process through which components/structures are produced layer-by-layer. In this context, 4D printing combines 3D printing with time so that this combination results in additively manufactured components that respond to external stimuli and, consequently, change their shape/volume or modify their mechanical properties. Therefore, 4D printing uses shape-memory materials that react to external stimuli such as pH, humidity, and temperature. Among the possible materials with shape memory effect (SME), the most suitable for additive manufacturing are shape memory polymers (SMPs). However, due to their weaknesses, shape memory polymer compounds (SMPCs) prove to be an effective alternative. On the other hand, out of all the additive manufacturing techniques, the most widely used is fused filament fabrication (FFF). In this context, the present paper aims to critically review all studies related to the mechanical properties of 4D-FFF materials. The paper provides an update state of the art showing the potential of 4D-FFF printing for different engineering applications, maintaining the focus on the structural integrity of the final structure/component.

## 1. Introduction

Additive manufacturing (AM) is a term attributed to the process of adding and joining materials, layer-by-layer, to fabricate objects. With this technological process, it is possible to create three-dimensional objects directly from CAD models [1]. Industrial/business machines, consumer products, and the automotive industry are the main fields where AM is used [2]. Despite that, there are markets where AM processes are becoming more and more popular, such as the medical field [3] (through the printing of living tissue [4]), aerospace, and the energy field. Some applications require a change in the behaviour or shape of the printed structure. Reducing the manufacturing time and reducing the manual labour required to assemble machines or objects are two of the many advantages of 3D- and 4D-printing. Additionally, 4D-printing reduces the time and effort required for logistics, transportation charges, and the volume of parts that can be transported [5]. Four-dimensional printing is defined as “3D printing + time”, where the characteristics/properties/features of the objects being manufactured (such as shape, properties, or functionality) can change as a function of time [6]. This time-dependent, printer-independent, and predictable technique allows the printed structures to achieve self-assembly, multi-functionality, and self-repair. Four-dimensional (4D) printing is a term that defines the incorporation of 3D printing and active material technologies [7].

In terms of 4D-printed parts’ properties (shape fixity, shape recovery, and repeatability), they are frequently attributed to the quality of the components. For example, one of the main characteristics of these parts is “repeatability”, which is the materials’ capability to repeat the entire cycle without cracking or without significant changes to the permanent shape [8]. The “shape fixity” is the extent of a temporary shape being fixed for a “shape memory polymer” (SMP) [9]. The ability of a 4D printing part to induce a mechanical deformation applied during the programming process is the “shape fixity ratio”. This parameter is calculated “from the ratio of the strain (ε unload) measured upon removal of the load after the cooling process, to the strain (ε load) detected at a specific temperature above the T_g_” [10,11]. Smart materials have the ability to give specific properties to the 3D models that are printed with them [12]. As a result, the printed components can respond to an external stimulus, changing their shape and/or volume and modifying their mechanical properties, such as Young’s modulus, stiffness, and strength [13,14]. In 4D printing terminology, folding, bending, twisting, linear or nonlinear expansion/contraction, surface curling, and the generation of surface topographical features are considered shape-shifting behaviours.

According to Zhou et al. [15], shape-shifting materials could be divided into two sub-classes: shape-changing materials (SCM) and shape-memory materials (SMM). A SCM is a material that has the ability to change shape instantaneously once a stimulus is applied and it can return to its permanent shape immediately after the stimulus is removed [6]. This type of transformation is limited to simple modifications as linear volume expansion and shrinkage [6]. However, the shape memory effect (SME) is a more complex transformation. SMM modifications involve two steps: the first one (the programming step) involves the deformation of the structure, which is maintained in a metastable temporary form; in the second one (recovery step), the structure can return to its original profile with an appropriate stimulus. Unlike shape-changing materials, shape memory materials can embrace a temporary shape until the right stimulus is applied. Therefore, SMM can be subdivided into two groups: the first one is “one-way shape memory materials”, for which the transformation is irreversible and a new programming step is needed to re-create the temporary shape after the original shape is recovered [15,16]. However, in the second group (“two-way shape memory materials”), the shape can be altered reversibly [15,16]. Although the investigation of this technique also points to health-related areas [12,17,18], this paper will focus only on studies related to the mechanical performance.

On the other hand, the technical literature reports that 4D printing techniques use the technology available for 3D printing if the printer is compatible with the materials used. Inclusively, it is reported that any comparison of 4D printing techniques will be a discussion of AM techniques [19], which are grouped by the American Society for Testing and Materials (ASTM) [20]. Regarding extrusion-based AM processes, the two most used methods are fused filament fabrication (FFF) and direct ink writing (DIW). The first is the most used technique to produce 4D-printed parts due to its simplicity, low cost of equipment, and raw materials (almost all thermoplastic materials), non-requirement of chemical post-processing, high speed, and large size capabilities. [20,21,22,23,24]. However, in addition to the high temperature required to melt the raw filaments that can have a detrimental effect on the mechanical properties of the parent material [25], its intrinsic accuracy and surface finish limit its applications in many respects [26]. In response to these drawbacks, the DIW technique has advantages due to the free choice of materials, small amount of raw material, open code, low cost, viability for multi-material printing, and construction of complex shape/structures without additional masks or dies [25,26]. Nevertheless, to achieve these benefits, polymeric inks must have good rheological and mechanical properties. In more detail, they should flow under high-strain conditions, but cease the flow or recover the mechanical integrity after extrusion. In this context, while the shear-thinning performance facilitates the extrusion of the ink, when under pressure in the nozzle, it should have a high shear elastic modulus and shear yield strength outside the nozzle due to the abrupt decrease in shear force [26,27]. Photopolymerization is another group in which the stereolithography (SLA) and digital light processing (DLP) are the most important techniques [21,25,28,29]. The main advantages are associated with a fast printing speed and excellent print resolution, although they are limited only to photocurable resins and confined essentially to heat stimulus [25,29,30]. Finally, the material jetting, also known as photopolymer inkjet printing or multi-jet modelling, is a liquid-based printing method that uses multiple materials to produce a component [31]. This technique uses multiple materials, such as composite shape memory polymers (SMPs) and composite hydrogels with widely adjustable properties; however, the main drawbacks are the cost of the printer, maintaining workability, coarse resolution, and the lack of adhesion between the layers [28]. Moreover, the stimulus to actuate 4D-printed structures is, usually, heat [25].

Therefore, in this context, this review will focus only on studies related to the mechanical properties and printing characteristics of 4D models printed via FFF that display SME when exposed to an external stimulus. For this purpose, the studies that support the present work focus on papers published in English, on scientific documents indexed in the Scopus database, combining “4D and FFF” or “4D and FDM”, and only papers related to mechanical performance, or those that provide sufficient information for the estimation of the material mechanical properties. Finally, the document is structured according to type of material (SME effect in polymers and SME effect in polymeric composites) and, regardless of the number of papers published, in each of these sections the studies are listed by year of publication and type of polymer. Lastly, because there are no differences between the fused deposition modeling (FDM) and fused filament fabrication (FFF) printing process, since the first is a registered trademark of the company Stratasys and the second is untrademarked, both terms can be used.

## 2. Shape Memory Effect in Polymers

Three-dimensional and four-dimensional printing techniques are very similar, but the advantage of 4D printing technology over 3D printing is the ability to produce objects that change their shape over time [7]. Therefore, the introduction of smart materials allows the 3D model to capture responses to external stimuli and use them for shape recovery, sensors, and actuators. In this context, for example, the 3D-printed structure may change its colour, shape, function, or other predefined characteristics in response to stimuli such as temperature, water, solvents, pH, light (ultraviolet rays), or magnetic energy [5,32]. From the smart materials with the shape memory effect, those most conducive to additive manufacturing are shape memory polymers (SMPs) [5,14]. They are macromolecular smart materials that react to an external stimulus, altering their macroscopic properties. Their main drawbacks are low stiffness and low recovery stress. Several reviews papers have been published on various SMPs, with special focus on thermal responsive ones [33,34,35,36,37,38,39,40,41,42]. However, regarding SMPs in 4D printing, there are fewer publications. In this context, the present section intends to summarise all the works published to date, in order to develop a critical review about FFF-4D-printed shape memory polymers, from the perspective of structural integrity and mechanical performance.

Goo et al. [43], for example, developed an efficient 4D printing method using the anisotropic thermal deformation of 3D-printed samples. This study used a fused filament fabrication (FFF) printer and an ABS filament. The temperatures of the nozzle, bed, and chamber were 240, 115, and 60 °C, respectively. The authors defined a layer thickness of 0.2 mm and a printing speed of 80 mm/s. In order to minimise in-plane anisotropy, it is generally recommended to use 45° and −45° raster angles alternatively. However, in this study, authors chose the raster angle intentionally to generate thermal anisotropy in a bidirectional manner (a longitudinal printing path with a 0° raster angle and a transverse printing path with a 90° raster angle). This was possible due to an effective combination of longitudinal and transverse printing paths. The thermal deformation after heat treatment was caused by the intentional anisotropy which contained residual stress. The modifications were caused by the contracting dimensions in the printing direction and expanding in the lateral and lamination directions. By studying the directional size changes of the homogeneously laminated specimens, authors investigated the relevant thermal deformation characteristics. Therefore, applying heterogeneous lamination, in which transverse and longitudinal printing paths were used consecutively, directional thermal deformation was used to enable samples’ 4D printing. After heat treatment, the heterogeneously laminated bars showed bending deformation, which corresponded to 1D-to-2D shape transformations. When compared to other 4D printing methods that use shape memory material or multiple materials, the proposed method uses a personal material extrusion (ME)-type 3D printer and a single thermoplastic polymer that does not have shape memory properties but promotes advantages in its usefulness and adaptability. It is also different from conventional 4D printing, in terms of its reversibility, because, while in most cases the shape transformation in 4D printing are temporary and reversible, the proposed method promoted permanent thermal deformation (after an appropriate heat treatment) for the printed parts. Therefore, for applications where permanent deformation is required, after thermal stimulation, these unique characteristics can be an advantage when used.

Zhang et al. [44] reported a self-healing 4D printing double network SMP system. They integrated a semi crystalline linear polymer (polycaprolactone (PCL)) as a healing agent into a methacrylate-based SMP system. Moreover, this system presents good compatibility with digital light processing-based 3D printing technology and can be used to successfully print complex high-resolution 4D printing models (up to 30 μm). In addition to that, these authors studied the effects of PCL concentration on the thermomechanical performance, viscosity, and self-healing capability of a self-healing shape memory polymer (SH-SMP) system. Computational fluid dynamics simulations were also carried out to investigate the effect of the SH-SMP solution’s viscosity on the printing process. It was concluded that, with the addition of more than 20 wt.% of PCL into the SH-SMP system, PCL linear polymer imparts the self-healing ability to the 4D printing structures, and the mechanical properties of a damaged structure can be recovered to more than 90%.

Hu et al. [45] introduced a 4D printing method to program SMP during the printing process. This method was applied by the FFF process, and it was used to print complex polymeric structures by self-bending features without the need for any post-programming. Tests were oriented to validate the feasibility of one-dimensional (1D)-to-2D and 2D-to-3D self-bending upon heating above the glass transition temperature. These authors concluded that, by heating, it is possible that the 3D-printed plate structures can be transformed into masonry-inspired 3D curved shell structures. Simultaneously, they also observed that, during the printing process, a good reliability of SMP programming was obtained. At same time, these authors developed a finite element (FE) formulation considering von Kármán geometric nonlinearity, which was solved by implementing an iterative Newton–Raphson scheme. This formulation was attached to a 3D macroscopic constitutive model for SMPs in order to realise the programming mechanism during fabrication and shape shifting during activation. This computational tool permitted the authors to accurately estimate the pre-strain induced in the fabrication and simulate 1D-to-2D and 2D-to-3D self-bending. Additionally, high reliability of the SMP programming was demonstrated experimentally and numerically.

Bodaghi et al. [46] were the first authors to use triple shape memory polymers (SMPs), using four-dimensional (4D) printing technology, and modelling adaptive structures for mechanical devices. The main approach used was related to the modification of SMP cold-hot programming using the fused decomposition modelling technology to design adaptive structures with triple memory effect (SME). Tests were carried out to characterise the elastoplastic and hyper-elastic thermo-mechanical properties of the material, as well as the regime of large deformation at low and high temperatures. It was noted that, for low temperatures, the printed SMP has an elastoplastic response while, for high temperatures, it behaves hyper-elastically in the regime of large deformation. Therefore, the possibility of printing and programming SMPs with dual and triple SME and self-bending feature was confirmed experimentally. For the quantitative understanding of dual/triple SME of SMPs printed by 4D in the large deformation range, they developed a phenomenological 3D constitutive model that incorporated crucial elements such as SMP phase transformation, hyper-elasticity, elastoplasticity, and hot–cold programming in a large deformation regime. A computational tool was developed to simulate dual/triple SMP structures using an algorithm developed for this purpose. The experimental and numerical results show a great potential for 4D-printed dual/triple SMPs mechanical applications.

Damanpack et al. [47] analysed the contact and impact behaviours of SMP beams printed with 4D technology via constitutive modelling, FEM formulation and simulation, as well as supported by experimental tests. They used a node-to-surface algorithm to evaluate the global/local contact and to simulate the visco-elastic-plastic behaviour of SMPs, while an innovative constitutive model was developed to predict the shape memory effect in large strain regimes. For this purpose, authors applied the Newmark and Newton–Raphson methods together with an iterative-incremental approach based on a visco-elastic-predictor visco-plastic-corrector return mapping algorithm to solve the equations that govern the FEM in spatial and time domains. The effects of substrate thickness, indentation location and edge effect were analysed, as well as validity of the Hertz theory for the load–displacement response of elastic materials. It was possible to conclude that, regardless of the temperature, the constitutive model was able to repeat elastic-plastic and hyper-elastic behaviours of SMPs. Simultaneously, for thin elastic substrates, the contact stiffness drops down drastically in the edge area, while it remains unchanged in the middle of the substrate. However, this variation becomes smooth with increasing thickness. The Hertz theory was not valid to be used in the edge area, and the results obtained with the models proposed for the maximum and residual beam displacement, impactor velocity, and forced-vibration configuration of the beam agree well with those obtained from the experimental tests for low-velocity impacts. No plastic deformation was produced by high-velocity impacts and the residual plastic deformation can be fully recovered by simply heating.

**Figure 1:**
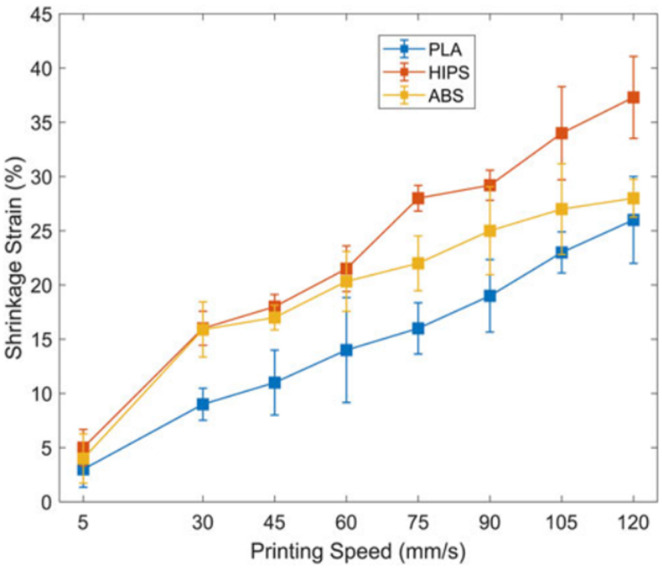
Effect of printing speed on the shrinkage strain [48]. Material from: Rajkumar, A.R.; Shanmugam, K, Additive manufacturing-enabled shape transformations via FFF 4D printing, published (2018) (Springer Nature) reproduced with permission of SNCSC.

Rajkumar et al. [48] reported the mechanisms that promote the thermally actuated shape-transformation in polylactic acid (PLA), high-impact-polystyrene (HIPS), and acrylonitrile-butadiene-styrene (ABS). Single-layer strips of different thermoplastics were tested at different speeds to assess the influence of printing speed on the shrinkage strain, and it was observed that higher printing speeds promote poor print quality. For samples printed at 120 mm/s, HIPS presented the highest shrinkage strains, followed by ABS and PLA, with average values of 37.3%, 28%, and 26%, respectively, while the lowest strains (less than 5%) were obtained for 5 mm/s. Figure 1 shows the shrinkage strain against printing speed (from 5 to 120 mm/s).

**Figure 2:**
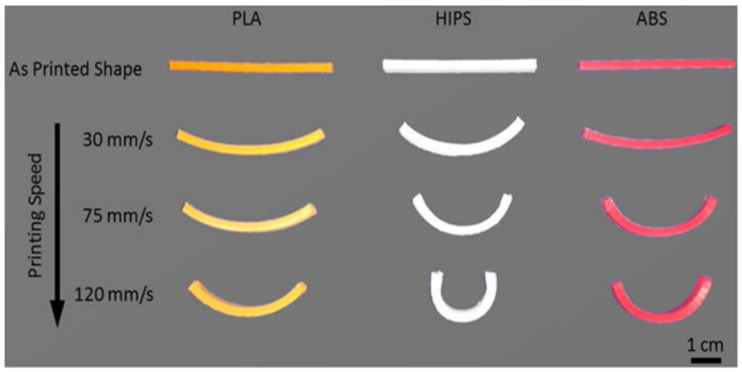
Effect of printing speed in different tested materials [48]. Material from: Rajkumar, A.R.; Shanmugam, K, Additive manufacturing-enabled shape transformations via FFF 4D printing, published (2018) (Springer Nature) reproduced with permission of SNCSC.

**Figure 3:**
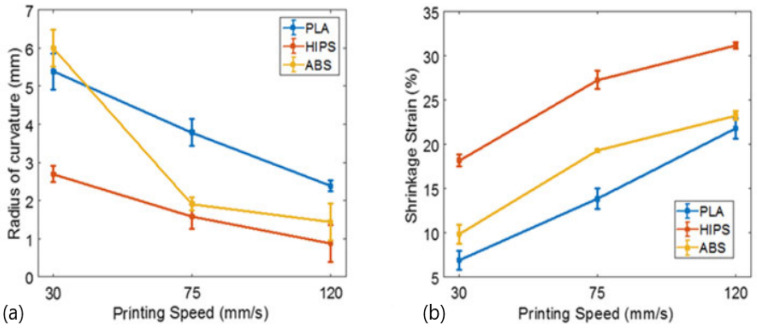
Effect of printing speed on the: (**a**) radius of curvature; (**b**) shrinkage strain [48]. Material from: Rajkumar, A.R.; Shanmugam, K, Additive manufacturing-enabled shape transformations via FFF 4D printing, published (2018) (Springer Nature) reproduced with permission of SNCSC.

For temperatures of glass transition temperature (T_g_) −20 °C, a significant decrease in modulus and strength was observed, while the failure strain increased. However, for temperatures of T_g_ + 20 °C, all samples had failure strains higher than 500%. On the other hand, there was a considerable decrease in stiffness, strength, and failure strain in samples printed at high printing speeds (105 and 120 mm/s). This was explained by the sparsity of the material deposited, resulting in an increase in the number of voids and a decrease in the adhesion properties with adjacent beads, leading to a decline in mechanical performance. However, while the mechanical properties decreased, higher printing speeds promoted an increase in shape transformation response. For example, PLA showed a recovery stress of 2 MPa for 105 mm/s, followed by ABS with a recovery stress of 1.72 MPa and HIPS with 0.95 MPa, both for 90 mm/s. In order to study the effect of printing speed on the shape transformation, these authors produced beam structures (2 mm thick) using the fused filament fabrication (FFF) method, and three different speeds (30, 75, and 120 mm/s) were considered. Figure 2 shows the self-bending action (from straight beam to curved beam), where the mid-plane shrinkage strain increases with increasing print speed, similar to the results shown in Figure 3 that were obtained for single layer samples.

**Figure 4:**
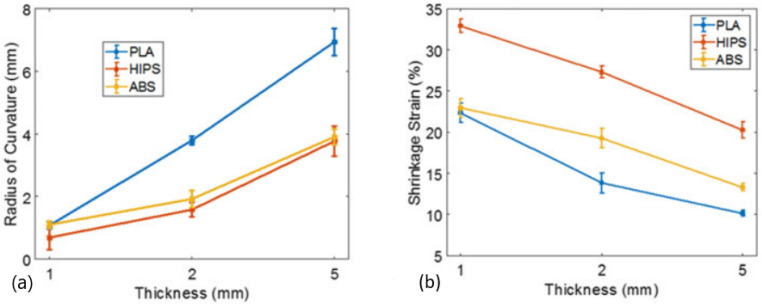
Effect of thickness on the: (**a**) radius of curvature; (**b**) shrinkage strain [48]. Material from: Rajkumar, A.R.; Shanmugam, K, Additive manufacturing-enabled shape transformations via FFF 4D printing, published (2018) (Springer Nature) reproduced with permission of SNCSC.

The effect of thickness (1, 2, and 5 mm) on the shape transformation was also analysed, considering a printing speed of 75 mm/s, and it was possible to observe that increasing the thickness increases the radius of curvature (R), while the curvature (1/R) decreases. As shown in Figure 4, the mid-plane shrinkage strain decreases with the increase in the thickness.

To study the effect of in-plane anisotropy on the shape transformation, circular discs for three different print orientations ([90°/0°], [0°] and concentric) were produced via FFF method with a printing speed of 75 mm/s. The authors concluded that, for samples with [90°/0°], the self-bending action is dominated by the print direction of the top layers. On the other hand, the curvature of bending is higher than that of unidirectionally printed discs. Simultaneously, when compared to unidirectionally printed discs, the dimensional changes are lower. Therefore, these authors concluded that increasing the printing speed promotes higher bending action, which is dominated along the print direction of the top layers. With a decrease in infill density, the distance between two deposited beads intensifies. In this case, the top layers can shrink with less constraint compared to discs with 100% infill density, and there was a significant dimensional change with the decrease in infill density.

Leist et al. [49] studied the shape memory behaviour of PLA and the properties by itself and when combined with nylon fabrics to create smart textiles. In terms of manufacture, two types of 4D printing were used: PLA deposited directly onto the printing bed; and PLA deposited directly onto the nylon fabric. For both cases, the printing speed was 100 mm/s and the nozzle temperature was 230 °C. To study the shape memory repeatability of the PLA, specimens were produced with a width of 10 mm, a length of 40 mm, and different thicknesses (800, 1000, and 1200 μm). Water heated at 65, 75, and 85 °C was used as a stimulus and the shape memory efficiency was assessed by the position angle. It was observed that, regardless of thickness, the mean angle of curvature increases with increasing temperature. Higher temperatures and thinner 3D-printed materials bring together the most suitable conditions for fast reacting smart materials and are more likely to return to their original permanent shape. For example, 800 µm-thick specimens immersed into water at 85 °C presented the highest shape recovery (around 75.5%), while the 1200 µm-thick specimens immersed into water at 65 °C reported the lowest shape recovery (about 42.9%). However, when the specimens were heated above their T_g_, they return to their permanent shapes in just seconds.

According to Wu et al. [50], the literature presents several studies reporting the influence of process parameters on mechanical properties, but their effects on the shape-memory are not fully understood. Therefore, they evaluated the influence of the raster angle (θ), deformation temperature (T_d_), layer thickness (H), and recovery temperature (T_r_) on the shape recovery ratio (R_r_) and maximum shape recovery rate (V_m_) of a polylactic acid (PLA) printed using a 3D printing technique. The shape recovery ratio is defined as the ratio of the difference between the original deformation and the recovery deformation, while the shape recovery rate is the recovery deformation by unit time. It was observed that recovery temperature, deformation temperature, raster angle, and layer thickness were the factors that, in descending order, had the greatest effect on the shape recovery ratio, and the optimal values to maximise this parameter were 70 °C, 55 °C, 45° and 150 µm, respectively. On the other hand, in terms of shape recovery rate, the order was recovery temperature, layer thickness, deformation temperature and raster angle, and the optimal values to maximise this parameter were 70 °C, 300 µm, 55 °C and 15°, respectively. For example, while the thickness of the layer had the smallest influence on the shape recovery ratio, it was responsible by a higher influence on the shape recovery rate. In terms of raster angle, this parameter has little effect on both variables, but the smallest effect was observed for the shape recovery rate. They also noted that the highest shape recovery ratio and maximum shape recovery rate were, respectively, 98% and 2.036 mm/s, but the shape memory effect depended heavily on the recovery temperature. Therefore, they concluded that parameter selection is crucial for 4D printing.

Wang et al. [51] produced a paper actuator by printing only a single layer of conductive polylactide (PLA) on paper using a FFF-3D printer. They reported that the main contribution of this study was the design of a composite that combined three physical phenomena: resistive electrical heating with conductive thermoplastic, the shape memory effect, and bi-layer actuation.

Bodaghi et al. [52] produced self-bending/morphing/rolling structures using 4D printing, and the effect of printing speed on the self-morphing characteristics was analysed. Considering a beam, they observed that lower speeds promote lower bending angles, and increasing the printing speed increases the bending angles, becoming like a conical panel. Therefore, the printing speed can be manipulated to obtain a desired angle. Simultaneously, these authors simulated the thermo-mechanical behaviours of structures (1D-to-2D and 2D-to-3D shape transformations) using the Abaqus software (HKS Inc., Rhode Island, USA), and good accuracy was observed with the experimental results.

Gu et al. [53] introduced a new approach for surfaces with self-rising continuous double-curvature textures. Geodesy consists of printing closed geodesic curves along the outline of a tile to form a thermoplastic sheet. The overall shape is achieved by transformation of the tiles into their own geometry. Authors implemented a design tool that was a simulator and a compiler. Due to the simulator, it was possible to see the approximate 3D geometry after morphing. In addition, this tool can assist the user in further informed modifications. Therefore, the authors made possible the customization and rapid prototyping of morphing surfaces.

Momeni et al. [54] established a new paradigm for the design and manufacture of wind blades using the 4D printing process. In this case, the designed blade can have a reversible bend-twist coupling (BTC) without relying on conventional electromechanical systems to change its shape in order to achieve the desired deflection. On the other hand, because the blades need to be flexible, the existing blades capable of BTC using passive methods have inherent flutter instability. However, with this new proposed blade, this problem has been overcome. It was also reported by these authors that some properties of materials printed by 4D (for example, the elastic modulus) are different from the moulded/annealed materials. Therefore, the real properties of printed materials should be used in the mathematical models used to predict the shape-shifting behaviour over time. Other benefits reported by the authors, in comparison with the conventional blade structure, is the fact that the proposed blade configuration has better structural and mechanical properties. Based on numerical simulations, for example, Liu et al. [55,56] showed that glass/carbon laminates are preferred to glass laminates for coupling design, providing a high coupling coefficient for off-axis fibre angles between 15° and 25°. Both the strains and maximum tensile and compression stresses increased with the increase in off-axis fibre angles; however, it was recommended to place the fibres with angles lower than 25° to ensure the blade structure safety. In terms of fatigue life, based on real environments (random wind loads, rain, snow, and self-weight), the best values were achieved on blades with ply angle between 45° and 65° that coincide with the side vein angle of most plant leaves. The same authors observed that, based on mathematical models, it is possible to orient the mismatch-driven forces into desired directions in order to provide the BTC shape shifting or, in other words, to print the active and passive materials. Therefore, in order to obtain the shape memory effect (SME), structures made of thermo-responsive shape memory polymers (SMPs) need both heat (thermal part) and an external force (mechanical part). In this case, using 4D printing, only heat is needed as a stimulus, because the force is obtained internally through active and passive materials.

Based on a negative Poisson’s coefficient structure obtained by 4D printing, Lin et al. [57] considered two types of personalised shape memory vascular stents. The optimised unit cell was obtained by means of a genetic algorithm and the three-dimensional models of the stents were obtained by bending the unfolded plane geometries with 21.4% (Type 1) and 21.1% (Type 2) surface coverage, producing the two types of structure analysed. For this study, shape memory PLA particles were extruded to produce printing filaments, and the models obtained by fused filament fabrication (FFF) on a 3D printer with 0.4 mm nozzle diameter, printing temperature of 190 °C and printing speed of 5 mm/min. In order to characterise the mechanical performance of the structures, and to ensure their practical applications, compressive and radial tests were performed. From the compressive tests, it was found that the maximum load and displacement were, respectively, around 57 N and 2.4 mm for the Type 1 stent and 53 N and 2.8 mm for the Type 2 stent. According to the authors, the better mechanical performance of the Type 1 stent is due to the larger size of the structural unit and, consequently, the smaller number of units. With respect to radial compressive tests, similar results were obtained, but the maximum load of the Type 1 stent, in this case, was two times higher than the value obtained for the Type 2 stent. Finally, in terms of three-point bending tests, they revealed that both structures present similar flexibility, and both recovery ratios reached about 98%.

Liu et al. [58] studied, experimentally and theoretically, nine different specimen configurations, combining straight line and polygon fill patterns manufactured by FFF, to explore the anisotropic effect on shape memory performance. PLA filaments were used and, after thermal characterization performed by the authors, values of 63, 110, 170 and 345 °C were found, respectively, for the glass transition temperature (T_g_), crystallization temperature, melting temperature, and degradation temperature. In terms of printing path, a straight line corresponding to five different directions ([0], [90], [±45], [0/90], [±60]), a triangle, and a hexagon (both corresponding to the directions of ([0], [90]) were considered in this study. From the experimental tests, the authors observed that the tensile behaviour of the filaments is characterised by a brittle behaviour, where the stress is nearly proportional to the strain until the ultimate stress (around 35 MPa). The tensile modulus and ultimate strain were, respectively, 627.1 MPa and 3.6%. Regarding the samples with different patterns, straight-line structures with [90] filling path showed the highest strength and modulus, with values around 1.72 and 1.32 times higher than those of the [0] filling path. These values evidence the low adhesive strength between printed filaments compared to the strength of filament itself. On the other hand, the authors concluded that triangular and hexagonal patterns have a lower effective modulus and strength than straight-line patterns, due to the internal voids of cellular patterns that induce lower density. In terms of compressive behaviour, hexagonal patterns showed higher strength and modulus than triangular patterns, regardless of whether the gradual failure phenomenon is observed for both configurations. Consequently, these patterns have a greater capacity for absorbing energy and, subsequently, it is possible to create a device with the purpose of absorbing energy, without the geometric borders and limitations of 3D printing. The authors also developed an analytical study based on the classic laminate theory and the honeycomb equivalent module theory, respectively, for straight-line and polygonal patterns, and the results obtained show good agreement with those obtained in the experimental results (reasonable error range). In terms of the relaxation modulus, it was observed that the modulus decreases dramatically over time, during the first 100–200 s, followed by a gradual decrease until reaching a stationary value as time approaches infinity. Comparing the different patterns, the straight-line pattern showed the smallest difference between the initial and final relaxation values (around 59.2%) after 1200 s, while the cellular patterns showed the greatest differences in the relaxation modulus due to the existence of internal voids. Finally, regarding the shape memory properties (SMPs), the printed samples showed a shape recovery ratio greater than 91%, but the straight-line specimens showed the lowest values, which decreased when the temperature increased from 55 to 65 °C. The shape fixity ratio was greater than 99.7%, after 20 h at room temperature, and the contraction rates of the 2D and 3D convertible structure with five elements in the longitudinal and lateral direction can reach values up to 40% and 18% (changing the length ratio and element number), respectively.

Mehrpouya et al. [59], using PLA filaments and the FFF technique, analysed the effect of total thickness (0.9–1.5 mm), layer height (0.15–0.30 mm), nozzle temperature (175–225 °C), and activation temperature (65–75 °C) on the shape recovery of origami structures. The samples were printed with 100% infilling density, a printing bed temperature of 40 °C, and printing speed and travel speed of 50 and 100 mm/s, respectively. The results were focused on the recovery ratio and unfolding rate. It was observed that an activation temperature of 65 °C, slightly above the PLA T_g_ (62–63 °C), promotes a recovery ratio around 85%, but when it increases to 75 °C, a full shape recovery is observed. On the other hand, when the minimum activation temperature (65 °C) was associated to the higher printing layer height, it was possible to maximise the shape recovery up to 90%. In this context, higher printing layer thickness, while the total thickness of the samples is constant, promotes fewer imperfections in the structure (air gaps) and, consequently, a greater chance of 100% of recovery ratios. The authors also observed a 10% increase in the recovery ratio when the nozzle temperature increased, within the recommended range, from 175 to 225 °C. Finally, they noted that the increase in the total thickness of the specimens from 0.9 to 1.5 mm promoted a recovery ratio 5% lower due to the higher accumulation of imperfections. In terms of unbending angle, the authors found that, for similar total thickness of the specimens, layer height had greater influence on the speed of unbending than on the activation time. Samples with the lowest layer height responded to the stimulus later than those with the highest layer height, which can be explained by having more layers and more air gaps and, in this case, they need more time for the heat to penetrate and affect the sample to react. Regarding the nozzle temperature, samples that were printed with higher temperatures react to the stimulus slightly later than those printed with lower temperatures, showing that this parameter presents small differences in the response time of the samples to the stimulus in the unfolding phase. Likewise, the total thicknesses can influence the activation time; however, the maximum recovery was reached almost at the same time. Finally, these authors observed that the activation temperature had a significant influence on the shape recovery of origami structures due to the higher heat transfer rate and lower viscosity of the PLA at higher temperatures. However, the heat penetration in the samples depends mainly on the structure instead of heat amount, while the effect on the shape recovery behaviour can be influenced by the amount of heat. Therefore, it was possible to conclude that, in decreasing order, the parameters that showed the greatest effect on the shape recovery response were activation temperature, total thickness, layer height, and nozzle temperature.

Mehrpouya et al. [60] investigated the effect of printing parameters on the shape recovery of sandwich structures with honeycomb core, with special focus on nozzle temperature, printing speed, and activation temperature. PLA filaments and a bed temperature of 40 °C, a layer height of 0.2 mm, and a raster angle of 0° were used as constant manufacturing parameters. The activation temperature was the water temperature used as a stimulus and, in this context, for higher temperatures, higher forces/moments are expected, and, consequently, a higher recovery ratio. According to the study developed by these authors, the average recovery ratio increased from 68% to 74%, when the water temperature varied from 65 to 85 °C. In terms of nozzle temperature, they observed that the recovery ratio increased from 62% at 190 °C to 68% at 210 °C, but for temperatures above 230 °C, no further change in the recovery ratio was observed. However, an inverse relationship was observed between the recovery rate and printing speed. On the other hand, in terms of unfolding rate, they noted that higher activation temperatures accelerated the recovery process, because higher temperatures cause faster heat absorbance in the samples. Regarding the nozzle temperature, samples printed with the lowest temperatures react to the stimulus significantly earlier than those printed with the highest nozzle temperature, because the melt viscosity of the PLA is strongly temperature-dependent and, consequently, has stronger interlayer adhesion. Finally, they concluded that, in terms of printing speed, samples produced with the lowest values react to the stimulus more quickly than those printed with higher printing speeds.

Noroozi et al. [61] used the 4D printing technology to design adaptive meta-structures which aim to control the propagation of elastic waves. Based on the SMPs’ thermomechanics, adaptive functionally graded (FG) beams were manufactured by 4D printing in order to mitigate vibration and acoustic attenuation. Experimentally and numerically, these authors demonstrated that 4D printing speed controls the shape recovery and self-bending capabilities of active elements. The FFF process was used to program shape-memory elements through layer-by-layer deposition, and the samples were tested to exploit 1D-to-2D self-bending capabilities as a function of printing speed. The samples were produced using polylactic acid (PLA) filaments with a glass transition temperature of 65 °C. Initially, the manufacturing parameters considered were: 0.1 mm thick printing layer, the printing raster was adopted along the length direction, temperatures of liquefier, build tray, and chamber around 210, 24 and 24 °C, respectively, and 5 mm/s printing speed to eliminate any pre-strains in the materials. These samples were used to perform DMA tests in axial tensile mode, ranging from 30 to 93 °C and with an applied dynamic stress around 1.5 times the static stress. It was observed that the increase in temperature promotes a decrease in the modulus, which drastically decreases the temperature to between 60 and 70 °C, and the glass transition temperature reduced to 65 °C. After this characterization, authors studied the effect of five different printing speeds (5, 10, 20, 40, 70 mm/s) on the shape recovery. Therefore, after printing, the samples were heated by immersion in hot water at 85 °C (20 °C above the transition temperature) and then cooled down to the room temperature. It was observed that at 5 mm/s, the samples did not change due to heating, but at 10 mm/s, there was a slight curvature after heating. Therefore, this printing speed was considered as a transient value due to an unbalanced pre-strain regime induced through the thickness direction during the printing process. Therefore, the increase in printing speed increased the bending angle and curvature, where the higher printing speed promoted greater pre-tension and, consequently, deformation. Finally, a 3D constitutive model was developed by the authors to estimate the effect of printing speeds on the shape recovery and self-bending of SMP beams printed by 4D. Simultaneously, they also noted that, by varying the printing speed and thermal excitation, the bandgap size and frequency range could be controlled and broadened using local resonances.

## 3. Shape Memory Effect in Polymeric Composites

In order to overcome the SMPs’ referred weaknesses, shape memory polymer composites (SMPCs) are being developed [32,62]. These materials are attractive because they combine the mechanical and functional properties, typical of the composites, with the shape memory properties. By using SMP matrices or integrating parts made of SMPs, such properties can be given to the composite materials and structures [63]. For metallic components, shape memory alloys (SMA) are smart materials that can directly convert thermal energy into mechanical work (a shape memory effect). Compared to SMA, SMP materials have many advantages, such high strain recovery, low density, cost, simple processing, biocompatibility and biodegradability [14]. Therefore, this section aims to summarise the papers published on 4D-printed SMPCs by FFF that showed a shape memory effect when an external stimulus was applied.

Nadgorny [64] printed a pH responsive polymer (poly (2-vinylpyridine) (P2VP), and it was noted that, by blending 12 wt.% of acrylonitrile-butadiene-styrene (ABS) with P2VP, the mechanical performance of the composite and processing performance was improved significantly. The 3D-printed samples show reversible pH-swelling properties. For example, the degree of swelling decreases with a higher degree of cross-linking and increases with quaternization by 1-bromoethane, but the addition of ABS considerably improved the mechanical stability of the P2VP, which was initially prone to damages caused by the 3D printing process.

Ly et al. [22], via FFF, confirmed experimentally the effect of printing parameters on polyurethane-based shape memory and on the same nano-enhanced polymer with carbon nanotubes (CNTs). After printing, the samples were immersed in a water bath and submitted to voltage experiments. The thermal response characteristic of FFF-3D-printed polyurethane-based SMP samples was maintained, but, due to the stimulus’ temperatures being higher than T_g_, the recovery times of the samples decreased significantly. However, in order to have an effective recovery time, the activation temperature must not exceed 10 °C below T_g_. On the other hand, higher printing temperature, filling ratio, layer thickness or lower feed rate promoted lower electrical resistance and faster recovery time.

Zhao et al. [65] observed that using FFF and a commercially available olefin ionomer (zinc-neutralised poly (ethylene-co-methacrylic acid)) makes it possible to obtain shape memory behaviour of 3D-printed samples. In addition, they compared samples printed in 3D and moulded by compression, noting that in the first ones, the initial recovery was lower (R = 58%) than in samples moulded by compression (R = 83%). The formation of polyethylene crystals that resist permanent network recovery was the cause of the poor recovery in the first cycle. However, due to the higher strain (lower modulus in the 3D-printed samples), for a fixed stress, this effect was improved in the 3D-printed samples. On the other hand, samples obtained by compression had lower recovery in subsequent shape memory cycles when compared to the samples printed in 3D. Finally, they concluded that with FFF as 3D printing technology, it is possible to obtain complex shapes of thermoplastic polymers with three-dimensional shape memory with an appropriate trigger (such as heat).

Kang et al. [66] produced and analysed a shape memory composite (SMC) which combined a shape memory alloy (SMA) with a shape memory polymer (SMP). SMA causes a shape memory effect due to the phase change between the martensite and austenite phases as consequence of the temperature change. On the other hand, SMP presents a memory effect promoted by changes in the proportions of hard and soft segments close to the glass transition temperature (T_g_). In this case, Nylon 12 (PA 12) was used as a 3D printing material in filament form. The base of the sample, which intends to simulate an actuator, was made by SMP, where two lines of nitinol wire were inserted. In the next step, the SMA was inset in the SMP part and fixed in the lines to form the SMC, which was covered by flexible PLA to improve its flexibility. Different volume fractions were studied and the optimum ratio (SMA:SMP of 1:5) promoted the largest length change (8 mm), and the most rapid response time (4 s) in overall dimension. On the other hand, when current flowed through the SMA wire, a reversible bending force was induced by the SME due to the Joule heating effect. For example, a current of 1.5 A flowing for 2 s was responsible for a moving distance of 7 mm with a temperature difference of 10 °C. By means of DSC and DMA analyses, these authors reported that, in a temperature range of 45–60 °C, the shape memory effect was perceptible. Finally, the reversible action mechanisms were investigated by tensile tests and they observed that, for low temperatures, SMP and SMA coexisted in fibre form, while the SMP changed to a matrix form for higher temperatures. Each single material showed low tensile strength, but, while for temperatures of 20 and 90 °C the SMA showed the smallest strain, SMP presented only a small strain at 20 °C and a significant strain at 90 °C. In terms of the bending modulus, values of 600 and 1180 MPa were found for SMA and SMP, respectively.

Peng et al. [67] produced polypropylene/nylon 6 (PP/PA6) filaments and observed that, by increasing the PA6 content, higher tensile and flexural properties of the printed samples were obtained, as well as higher dimensional stability. For example, tensile strength increased from about 13 MPa for neat PP to about 25 MPa when 30 wt.% of PA6 was added to PP. A similar increase was observed for bending strength, while the bending modulus increased from about 500 to 700 MPa (considering the same PA6 content). In addition to the PA6 content, these authors also analysed the deformation temperature (T_d_), infill orientation, and infill density of the samples. They found that 3D-printed specimens with 30 wt.% of PA6 manufactured by the infill orientation of 45°/−45° and 100% infill density have appropriate shape memory performance when the deformation temperature (T_d_) is 175 °C. Furthermore, the infill orientation that had the highest tendency to align with the applied force exhibits the lowest shape fixity (R_f_) and highest shape recovery (R_r_), but the infill density has little influence on such factors.

Kabir et al. [68] studied the shape memory and the tensile properties of 3D-printed composites (nylon fabric and shape memory thermoplastic polyurethane) for different thicknesses (0.2, 0.4, 0.6, 0.8, and 1.0 mm) and number of cycles (50 shape memory cycles). They concluded that, regardless of thickness, the composite had a 100% shape recovery ratio up to 50 cycles. In terms of shape recovery rates, this property remained similar from cycle 1 to cycle 50, but gradually decreased (from 3° to 0.7°/s) with the increase in the thickness. During the shape recovery process, the highest rate occurred in the intermediate stage, while the slowest occurred in the initial and final stages. Finally, the response time and thickness confirmed that a thicker specimen took more time, and a thinner specimen took less time to respond or underwent an initial recovery. The tensile properties were also investigated to assess the effect of the cycles number, and it was noticed that both stress and initial modulus of the composite increased with the increase in the number of shape memory cycles. Therefore, combing the advantages of excellent shape memory properties and good mechanical properties, this composite has potential for use in protective clothing that requires repetitive shape changes.

Liu et al. [69] studied multi-responsive shape memory polycyclooctene (PCO) and PCO composites with hexagonal boron nitride functional fillers (from 5 to 20 wt.%). Posteriorly, the samples were crosslinked by γ-ray irradiation (c-PCO and BN/c-PCO). It was possible to conclude that the incorporation of BN fillers with high thermal conductivity improved the print quality and the thermal response of SMPs. At the same time, the printed samples produced with BN/c-PCO composites showed better shape memory effect and higher recovery rate compared to pure c-PCO. In the same study, these authors also prepared multi-walled carbon nanotube/polycyclooctene nanocomposites (MWCNTs/PCO), and they found that increasing the fillers content increases the storage modulus and the electrical conductivity of the composites. For example, considering the higher filling content (20 wt.%) the storage modulus increased from 383 to 1454 MPa for −50 °C and from 1.63 to 18.7 MPa for 60 °C, while the electrical conductivity reaches values around 6.5 S/m. Therefore, the addition of MWCNTs endows PCO with higher conductivity and lower T_m_, enabling an electro-response for very low voltage values (5 V). Therefore, adding MWCNTs into PCO led 3D SMP devices to have a multi-responsive capability due to the improved electrical conduction and light absorption of composite materials.

**Figure 5:**
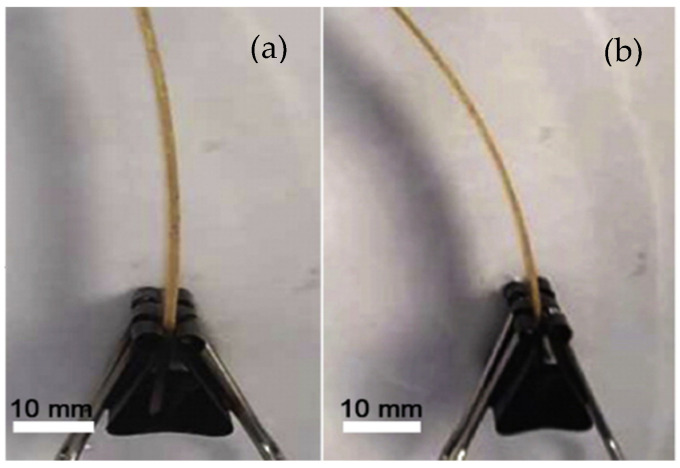
Hygromorph biocomposite produced by fused filament fabrication (FFF): (**a**) before and (**b**) after immersion in water [70].

Le Duigou et al. [70] used dispersed pinecone seeds and the hygroscopicity of the wood fibre to produce 3D-printed specimens, which allowed for the possibility of being activated by moisture (see Figure 5). Regarding the printing parameters, the nozzle temperature and the heating plate were 210 and 70 °C, respectively, and the printing speed was 18 mm/s, while the fibre content was around 15.2 ± 0.9% wt.%.

These authors reported that these printing parameters did not influence the wood biocomposites’ properties. The sample width presents an important effect on the biocomposites’ properties, because it controls the overlap of filaments and, consequently, the internal cohesion of the printed specimen. This parameter varied from 100% to 300%. The samples were printed following a rectilinear filling pattern oriented at 0° (longitudinal) or 90° (transverse) along the x-axis without any contour. The tensile tests were performed on 0° and 90° samples with three layers (≈1 mm) in dry and water-saturated states. To understand how the printing process affects the tensile behaviour and properties of biocomposites reinforced with wood, authors tested the filaments before and after printing. They observed a very similar behaviour for both, where, until the brittle rupture, an almost linear elastic behaviour was verified. For samples printed at 90°, authors observed that there was no change in the brittle behaviour; however, compared to the longitudinal ones, the stiffness and tensile strength decreased by around 20% and 35%, respectively. It was possible to conclude that the orientation of the wood fibre in the polymeric matrix was caused by the extrusion process (to produce filaments) and not by the FFF process itself. Compared to the compressed samples, the porosity measured in the printed samples was very high. For samples obtained with printing widths of 100%, 200%, and 300% (in the 0° direction), for example, an increase from 14.7 ± 1.4% to 15.5 ± 2.9% and 21.8 ± 1.2% was observed, respectively. When compared to the 0° printing direction, the samples printed along the 90° direction showed lower porosities (decreased to values of 8.4 ± 1.7%, 11.1 ± 3.1% and 14 ± 1.2% for print widths of 100%, 200% and 300%, respectively). On the other hand, in contrast to samples printed at 90°, samples printed at 0° exhibit a more ductile behaviour with increasing printing width, where non-linear behaviour was observed between stress and strain until rupture. For biocomposites prepared with 100% and 300% printing width, the tensile strength decreased around 30% and 50% when compared to compressed samples. In addition, the tensile modulus decreased by around 50% and 65%, respectively, which can be explained by the microstructure produced by 3D printing. In this case, the damage was caused by the significant porosity that was induced, which drastically affects the mechanical properties. It was also reported that samples of biocomposites produced by FFF exhibited lower mechanical properties than those manufactured by extrusion, compression, or injection, but greater hygroscopic sensitivity. Although the 4D concept was first defined in 2014 [71], there is still a lot of information about the correlation between FFF and the actuation properties induced by natural fibre composites. However, this study showed that the FFF process provides materials with adequate characteristics for a range of moisture-induced actuation functionalities in biocomposites, and with better mechanical properties.

Kačergis et al. [72] studied 3D-printed multi-material shape-variable structures imitating a hinge. Using the FFF process, different printing parameters were analysed. It was noted that 3D-printed structures remained flat after printing and until they were exposed to a stimulus (heat), but the influence of printing speed, temperature of the build plate, and the number of active layers in the structure was notorious. For example, when printing structures on a cooler surface and selecting higher printing speeds, it was observed that the deformation angle can be programmed. In fact, these two FFF printing parameters are responsible for the higher residual stresses stored in the samples, and this concept can be implemented in structures with more active layers to bend even further after the recovery step. The highest overall stiffness is maintained through this concept. On the other hand, they noticed that structures with more layers required more time to reach their final shape, because the inner layers did not reach the rubbery state and tried to return to the high entropy state as quickly as those located on the outer regions. Therefore, the number of active layers can be used for a sequential fold of a complex layout model, and multi-material morphing structures can be designed by changing the printing parameters. Finally, the authors believed that the hot water used as a stimulus to activate the structures can be replaced by other sources of direct and indirect heating.

Liu et al. [73] studied the microstructural design, shape recovery behaviour, and recovery force behaviour of 4D-printed angle-ply laminated and rectangular braided samples. For this purpose, they used carbon nanotube/polylactic acid (CNT/PLA)-based shape memory polymer (SMP) filaments. It was possible to conclude that, for the same volume fraction, the recovery force and flexural properties of angle-ply laminated samples were superior to those obtained with rectangular braided ones. Nevertheless, the same comparison in terms of shape recovery rate showed lower values for the angle-ply laminated specimens. On the other hand, when the PLA matrix was filled by CNT, there was an early initiation of the specimen shape recovery process. For example, depending on the configuration of the specimens, the recovery force increased by between 123% and 144%, and the peak load by around 28.5%. Conversely, the displacement at ultimate failure was smaller. The same authors, in another study [74], investigated the synergistic effect using, for this purpose, a spring steel strip (SSS) and a 4D-printed thermoplastic SMP. To optimise the shape recovery properties, different stacking sequences, filling percentages (50%, 75% and 100%), and SSS thicknesses were analysed [76]. In terms of recovery time, after heating, there was a decrease for lower SMP infill percentages due to less mass and larger heat conduction. For example, the recovery time observed for SMP/SSS specimens with 50% infill was reduced by 39% when compared to SMP samples. On the other hand, the recovery time of SSS/SMP specimens decreased for higher SSS thicknesses, because higher elastic energy is stored. The reusable reliability of hybrid composites was also demonstrated by analysing the free shape memory behaviour of the samples over five consecutive heating–cooling cycles and, for each cycle, the hybrid samples almost recovered their original configuration and the SSS–SMP interface did not show degradation. Therefore, the reliability of the synergistic effect on the shape recovery was confirmed. In terms of recovery force, these authors observed benefits around 198 times and 137 times higher when the recovery forces of SMP/SSS and SSS/SMP specimens with 50% infill and 0.2 mm SSS thickness were compared to SMP samples, respectively. At the same time, they observed a dominating influence of the SSS thickness on the recovery force, because higher SSS thicknesses promoted higher recovery force [74].

**Figure 6:**
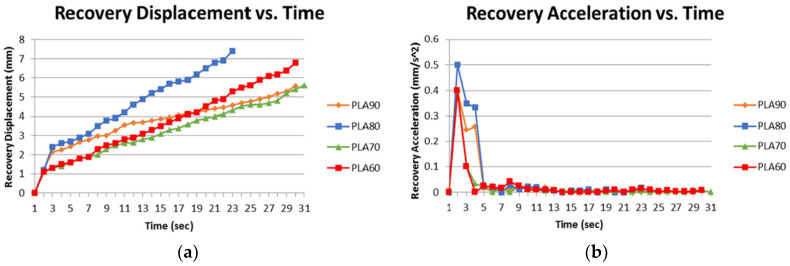
(**a**) Dynamic recovery displacement against time; (**b**) dynamic recovery acceleration against time [75].

A low-cost temperature-sensitive memory polymer for 4D printing, by the FFF process, was developed by Carlson et al. [75], as well as its kinetic evaluation. For this purpose, filaments were produced, by the extrusion process, with polylactic acid (PLA) and thermoplastic polyurethane (TPU) with different weight contents (60%/40%, 70%/30%, 80%/20% and 90%/10%). It was noted that after being exposed to temperatures above the glass transition temperature of the materials, the specimens showed fixity and shape recovery. From all materials, filaments produced with composites of 80%/20% (80% of PLA and 20% of TPU) showed the most consistent and best shape memory characteristics. This material can maintain good shape fixity and shape recovery ratios, as well as withstand repeated deformation and recovery cycles. Therefore, as shown in Figure 6, this material has the fastest recovery time.

Fu et al. [76] manufactured an intelligent switch printed with flexible PLA and semi-crystalline polymer PCL, in a double spiral structure, to verify the range of applications achievable in the field of smart devices (for example water valves). It was demonstrated that the intelligent switch implementation rules can be applied as black box rules in the fields of encryption and security. Furthermore, the stability of the temporary state of samples printed with different configurations was tested and optimised. In this context, when comparing the selected printed structures with the common shape-memory materials, the fixity ratio of the samples should be optimised for values higher than 88%, in order to achieve the desired performances. This requirement was achieved because, from the experimental tests carried out with specimens produced with dual material (PLA and PCL), the fixity ratio reached values up to 96.92%.

Pandey et al. [77] analysed the shape-memory effect, wettability, and biocompatibility analysis of a fused filament fabricated chitosan (CS)-reinforced polylactic acid (PLA)-based porous scaffold. For this purpose, PLA composites filled with 1, 1.5, and 2 wt.% of CS were produced by twin-screw extrusion. To make the shape memory effect (SME) feasible, the samples were treated by direct heating at 60–70 °C. It was possible to conclude that with increasing the CS content, the shape recovery decreased significantly because the CS particles act as rigid phases and interrupted the re-ordering of PLA chains during the printing. At the same time, they also noticed that the process parameters significantly influence the shape recovery. For example, while the infill density proved to be favourable to the shape recovery, the robustness of the scaffolds enhanced the stiffness and elasticity. Regarding the stimulating temperature, its increase increases the shape recovery characteristic of the printed scaffolding. From the wettability analysis, the authors observed that, after exposure to higher stimulating temperatures, the hydrophilic nature of the scaffoldings increased. Therefore, by increasing the CS content, porosity and temperature stimulus, the biocompatibility of the printed scaffolds is increased.

Using a preload and reverse stiffness combined with shape memory effects, Song et al. [78] suggested a method to generate the reconfiguration of mesostructures producing programmable deformations at high temperatures. Basically, the functional design of mesostructures is based on the exchange of deformation energy between two materials (reverse effect of stiffness with temperature). In this case, the recovery of the original shapes can be aided by an additional shape memory effect (stress relaxation of each component at high temperatures). The authors concluded that, unlike the conventional reconfigurable method with SMP, the reverse stiffness method has the potential to design reconfigurable structures with two thermal cycles (transformation and recovery) combined with preload at room temperature. In this context, structural materials with a negative thermal expansion and an order of magnitude higher than that of available metamaterials can be designed for specific locations of structures with the effect of reverse stiffness. They concluded the study with a design guideline for the thermo-mechanical transformation of mesostructures through phase maps, as well as the optimum geometric requirement for a pair of materials.

More recently, Yu et al. [79] used thermoplastic filaments to print 4D samples. This composite structure design, involving two materials (polylactic acid (PLA) and carbon fibre-reinforced PLA (CFPLA)), was based on FEA modelling and simulation to achieve the self-morphing behaviour. The 3D-printed samples were manufactured with three different printing parameters (printing path orientation, printing layer thickness, and filament material property) and, in order to correctly conduct the simulation, tests were performed to quantify the hyperelasticity and viscoelasticity of the thermoplastic component. For this purpose, uniaxial tensile and compression tests were performed to obtain the elastic component, and DMA tests were performed for the viscous component and uniaxial discharge–recharge with different stress levels for plastic deformation and the Mullins effect. Regarding the effect of the printing parameters, the authors found that PLA samples with a printing layer thickness of 0.1 mm have the best bending performance, while for 0.5 mm an inadequate bending performance was observed. On the other hand, all CFPLA samples showed minimal bending angles, indicating that the shrinkage ratio along the printing direction of the CFPLA is generally lower than that of PLA. In terms of flexural properties, CFPLA samples have the highest flexural stiffness; however, it is so fragile that it can break within a relatively small deflection range. On the other hand, the PLA sample can achieve large deflections, but it has less flexural stiffness. Therefore, when PLA and CFPLA were combined into a bi-layer structure, this system exhibits, at the same time, high flexural stiffness and great deflection.

## 4. Summary of the Shape Memory Effect

A review of all published works was presented in the previous sections, where the structural integrity and mechanical performance of materials printed by 4D via FFF was the main goal. At the same time, their behaviours were discussed together with the external stimuli that led to the change in shape. In this context, the present section aims at summarizing the main conclusions obtained in each work about shape memory polymers (Table 1) and shape memory polymer composites (Table 2).

From the present review, it is evident that 4D-FFF printing has a high potential in a large spectrum of engineering applications. At the same time, it seems clear that additional efforts should be done to better characterise and understand the mechanical behaviour (fracture and fatigue behaviour) of 4D-FFF printing components. In the authors’ opinion, this the key for the wide use of this process in a vast number of interesting and relevant engineering applications.

## 5. Conclusions

The scientific community shows a particular interest in 4D printing, because it allows structures to change shape over time, from a pre-programmed shape, in response to an external stimulus such as pressure, temperature, wind, water, and light. The intelligent materials, in this case, arouse enormous interest due to their intrinsic characteristics. When combining these two purposes, the self-transforming structures appear, which arouse the interest of several industrial sectors, such as medical, defence, and aerospace, among others. In addition, 4D printing can be specifically targeted to consumer needs, very particular designs, or consumer goods.

From this review regarding FFF-4D-printed shape memory polymers, it is possible to conclude that most studies focus on conceptual designs of such materials when exposed to different stimuli, but, unfortunately, very few studies can be found that address the problem from a structural point of view. In fact, the various studies reported in the technical literature analysed the effect of process parameters on mechanical properties or on shape memory, but, in both cases, they were not yet developed enough to have a complete knowledge of this subject and possible structural applications. For example, the viscoelastic and temperature-sensitive properties of SMPs and SMPCs should be analysed, as well as the effect of interfacial damage on the effective properties of SMPCs under different temperatures and large deformations. Therefore, a more comprehensive knowledge of the properties of SMPs/SMPCs is needed, which will help us to use them more effectively and rationally. For this purpose, more studies are expected for this technology to be applied in the most diverse industrial fields.

While, clearly, the use of 4D-printed materials is very promising, the detailed review of the state of the art summarised in the present paper suggests that more work needs to be done in this area in order to make this technology suitable for use in situations of practical interest.

Based on the knowledge gained via the present study, our recommendations for future research work/developments are summarised in the following bullet points:

- Systematic investigations aiming at creating robust links based on rigorous statistical tools between manufacturing variables and mechanical properties before/after external stimuli;
- Multi-variable experimental/theoretical investigations aiming at modelling the interactions amongst manufacturing process envelopes, mechanical properties, and effects of combined, time-variable external stimuli;
- Clear guidelines on how to design specific material lay-ups in order to manufacture components characterised by tailored mechanical behaviours, with the mechanical response being considered both in the presence and in the absence of the external stimuli of interest;
- Defining specific manufacturing rules suitable for choosing/optimising the printing directions according to the target mechanical properties of the objects being manufactured;
- Understanding the existing interactions between manufacturing process, mechanical properties, and effect of localised stress concentration phenomena;
- Exploring the above aspects not only in the presence of static loading, but also in the presence of dynamic and fatigue loading.

## Figures and Tables

**Table 1:** Studies related to shape memory polymers printed via FFF.

Year	Author	Polymers	Main Conclusions	Reference
2020	Goo et al.	ABS	Using the 4D printing method, it was possible to introduce localised bending deformation by laminating isotropic and anisotropic regions selectively, enabling a self-assembly function.	[43]
2019	Zhang et al.	Polycaprolactone (PCL)	With the addition of more than 20 wt.% of PCL to the created system, the linear polymer imparts the self-healing ability to 4D-printed structures, and the mechanical performance of a damaged structure can be improved by more than 90%.	[44]
2017	Hu et al.	Polyurethane (PUR)	The pre-strain induced in fabrication was estimated and the 1D-to-2D and 2D-to-3D self-bending was simulated. The high reliability of SMP programming was demonstrated experimentally and numerically.	[45]
2018	Bodaghi et al.	Polyurethane (PUR)	Triple SMPs by 4D printing technology and shape adaptive structures with self-bending feature were explored. Potential applications were demonstrated via experimental and numerical results.	[46]
2020	Damanpack et al.	PUR	Significant residual plastic deformation can be recovered by simply heating.	[47]
2018	Rajkumar et al.	Polylactic acid (PLA), high-impact-polystyrene (HIPS), acrylonitrile-butadiene-styrene (ABS)	HIPS present higher shrinkage strains followed by ABS and PLA, with average shrinkage strains of 37.3%, 28%, and 26%, respectively. While the mechanical properties decrease, at higher printing speeds there was an increase in shape transformation response. PLA showed the largest recovery stress (2 MPa for 105 mm/s), followed by ABS (1.72 MPa for 90 mm/s) and HIPS (0.95 MPa for 90 mm/s). When increasing the sample’s thickness, the radius of curvature (R) increases, while the curvature (1/R) and the mid-plane shrinkage strain decreases. On the other hand, the self-bending action is dominated by the print direction of the top layers.	[48]
2017	Leist et al.	Polylactic acid (PLA)	PLA was shown to have a thermal shape memory behaviour and these abilities were maintained when combined with nylon fabric. Both materials can be programmed into temporary shapes and return to their permanent shapes when heated.	[49]
2017	Wu et al.	Polylactic acid (PLA)	The optimal values for maximizing the shape recovery ratio and shape recovery rate depend on the deformation temperature, recovery temperature, raster angle, and layer thickness. In the study developed by the authors, the highest shape recovery ratio obtained was 98%, and the maximum shape recovery rate around 2.04 mm/s.	[50]
2018	Wang et al.	Polylactic acid (PLA).	Development of a composite and its manufacture, which combined three phenomena: electrical resistive heating of conductive thermo-plastic, shape memory effect, and bi-layer actuation.	[51]
2019	Bodaghi et al.	Polylactic acid (PLA)	Complex structures with self-bending/morphing/rolling features fabricated by 4D printing technology were analysed. Their thermo-mechanical behaviours using a simple computational tool were replicated. Using a comparison study with experiments and numerical results obtained from an in-house FE solution, a good precision of the proposed method was observed.	[52]
2019	Gu et al.	Polylactic acid (PLA)	It was created novel path planning approach for printing thermoplastics to form a surface with one raised continuous double-curvature tiles when exposed to heat. In the Geodesy tool, the authors implemented a design tool which visualises the approximated 3D geometry after morphing and further assists the user in making informed modifications.	[53]
2019	Momeni et al.	Polylactic acid (PLA) as active material Membrane of paper as passive material	Using the 4D printing process, a new paradigm for the design and manufacture of wind blades was created. The designed blade can have a reversible bend-twist coupling (BTC) without relying on conventional electromechanical systems to change its shape and to achieve the desired deflection.	[54]
2020	Lin et al.	Polylactic acid (PLA)	It was demonstrated that 4D-printed shape memory stents with negative Poisson’s ratio structure are highly promising for the treatment of vascular stenosis.	[57]
2020	Liu et al.	Polylactic acid (PLA)	The printed material showed a shape recovery ratio of more than 91% with the presence of water resistance and shape fixity ratio of more than 99.7% after 20 h at room temperature.	[58]
2020	Mehrpouya et al.	Polylactic acid (PLA)	Basic origami structure can be used for different purposes, such as: delivery or transplantation of biomaterials, grasping objects and plugging the defects. According to the authors, smart grippers, switches, sensors or actuators can be manufactured using the same strategy.	[59]
2020	Mehrpouya et al.	Polylactic acid (PLA)	It was reported that the optimal recovery ratio can be achieved employing higher activations and nozzle temperatures and lower printing speed.	[60]
2020	Noroozi et al.	Polylactic acid (PLA)	The printed models that have been tested have the ability of controlling elastic wave propagation through the formation of bandgaps or frequency ranges where the wave cannot propagate. The bandgap size and frequency range can be controlled by varying 4D printing speed and thermal excitation.	[61]

**Table 2:** Articles published on shape memory composites printed via FFF.

Year	Author	Polymers	Main Conclusions	References
2016	Nadgorny	12 wt.% of acrylonitrile-butadiene-styrene (ABS) with (poly (2-vinylpyridine) (P2VP)	The degree of swelling decreases with a higher degree of cross-linking and increases with quaternization by 1-bromoethane (BE). The addition of ABS has considerably improved the mechanical stability of P2VP.	[64]
2017	Ly et al.	Polyurethane reinforced with carbon nanotubes (TPU/CNT)	The thermal response characteristic of the FFF 3D-printed polyurethane based SMPs samples was retained when the specimens were placed into the water bath and were subject to voltage experiments. Because the stimulus temperatures were higher than T_g_, the recovery times of the specimens decreased significantly. The sample that had the highest printing temperature, filling ratio, layer thickness or lowest feed rate will exhibit lower electrical resistance and faster recovery time.	[22]
2017	Zhao et al.	Commercially available olefin ionomer (zinc-neutralised poly(ethylene-co-methacrylic acid))	A commercially available polymer can be used to print samples via FFF without any new chemistry requirements. The 3D-printed samples’ initial recovery was lower than that obtained by compression. The compression-moulded part had an inferior fixity and recovery in subsequent shape memory cycles, when compared to the 3D-printed part.	[65]
2018	Kang et al.	Nylon 12	A reversible bending force caused by a Joule heating effect was induced by the shape memory effect that occurs when current flows in the SMA wire. For lower temperatures, both SMP and SMA existed in fibre form, which increased the strength of the SMC. At higher temperatures, the SMP phase changed to a matrix form and a continuously reversible bending force is showed on the composite.	[66]
2019	Peng et al.	Polypropylene/nylon 6 (PP/PA6)	As the PA6 content increased, tensile and flexural properties of the printed samples gradually increased. Three-dimensionally-printed product based on PP/PA6 configuration with 30 wt.% of PA6 exhibited higher dimensional stability and adequate mechanical properties in FFF. Three-dimensionally-printed specimens with 30 wt.% of PA6 fabricated by the infill orientation of 45°/−45° and 100% infill density have appropriate shape memory performance when the deformation temperature was about 175 °C.	[67]
2020	Kabir et al.	Shape memory thermoplastic polyurethane (SMTPU) Nylon	The developed composite has considerable potential as a smart, reinforced and protective clothing that needs complex three-dimensional shapes.	[68]
2020	Liu et al.	Boron nitride/polycyclooctene (BN/PCO)	Incorporating high thermal conductive BN fillers into PCO improves the print quality and thermal response speed of SMPs. Better shape memory effect and higher recovery rate were obtained compared to neat PCO. Higher multiwalled carbon nanotube (MWCNT) content increases the storage modulus and electrical conductivity of the composites. Therefore, the addition of MWCNTs has led 3D SMP devices for multi-responsive capability due to the improved electrical conduction and light absorption of composite materials.	[69]
2016	Duigou et al.	Polylactic acid (PLA) and poly (hydroxyalkanoate) (PHA) matrix, reinforced with recycled wood fibre	The highest tensile stress obtained was around 30 MPa for composites with 0° fibre orientation. Water uptake ratio increases with reducing tensile strength.	[70]
2019	Kačergis et al.	Polylactic acid (PLA) and polyurethane (TPU) composite	The deformation angle can be obtained by printing structures on a cooler surface and selecting higher printing speeds, because these two parameters promote higher residual stresses stored in the samples. Structures with more layers required more time to reach their final shape.	[72]
2019	Liu et al.	Carbon nanotube/polylactic acid (CNT/PLA) and CNT/PLA/silicone	With the same fibre orientation and volume fraction, the recovery force and flexural property of angle-ply laminates were superior to those of rectangular preforms. The shape recovery rate of angle-ply laminates was lower than that of the rectangular preforms. Recovery force, flexural load and flexural modulus of 4D-printed composites were improved with the addition of CNT and silicone matrix to the preforms.	[73]
2019	Liu et al.	Polylactic acid (PLA) and carbon nanotube/polylactic acid (CNT/PLA)	The final shape times of the samples decreased with the reduction in SMP infill percentage. The final recovery time of the SMP/SSS specimen with 50% infill was reduced by 39% compared to the SMP specimens. SSS thickness is an important factor in improving recovery force.	[74]
2020	Carlson et al.	Polylactic acid (PLA) and polyurethane (TPU)	When PLA and TPU load is 80% and 20% of the respective weight, the filament showed the most consistent and best shape memory characteristics, maintains good shape fixity and shape recovery ratios, as well as resisting repeated deformation and recovery cycles. PLA has the ability to accelerate and maintain velocity during recovery.	[75]
2020	Fu et al.	Polylactic acid (PLA). Polycaprolactone (PCL)	The proposed low-cost, concealed, and intelligent switch can be applied in many fields, specifically in cases where smart controls without electric circuits are required. The device can be used as a black box for security and encryption applications.	[76]
2020	Pandey et al.	Chitosan (CS) Reinforced Polylactic acid (PLA)	As rigid phases, CS particles interrupted the reordering of PLA chain. The statistical analysis showed that loading of CS, infill density, and stimulating temperature contributed 31.59%, 61.72%, and 6.54% in the shape recovery factor. At optimised process parametric settings, scaffoldings demonstrated 18.8% shape recovery. The stimulated samples acquired good wettability and cell proliferation.	[77]
2020	Song et al.	Polyurethane (TPU) and Polylactic acid (PLA)	The recovery of the original shapes can be helped by an additional shape memory effect, by stress relaxation at high temperatures.	[78]
2020	Yu et al.	Polylactic acid (PLA) and carbon fibre reinforced PLA (CFPLA)	A computational workflow using FEA to produce physically accurate results of the residual stress-induced morphing behaviours of mesh-like thermoplastic was established. This work was validated the FEA modelling with an accurate matching between simulation and experiment for three examples.	[79]

## Data Availability

Data sharing is not applicable to this article.
